# Supplementary material for: Identification and characterization of in vitro expanded hematopoietic stem cells
Source: EMBO Rep. 2022 Aug 16;23(10):e55502. doi: 10.15252/embr.202255502 (PMC9535767; doi:10.15252/embr.202255502)

Appendix Table of Contents

Appendix Figure Legends 2

Appendix Figure S1 3

Appendix Figure S2 4

Appendix Figure S3 5

Appendix Figure Legends

Appendix Figure S1: Gene expression differences between NegNonELSK and PosNonELSK cells.

(A) Volcano plots showing differentially expressed genes between NegNonELSK and PosNonELSK cells (cutoffs: p-val = 0.05 and logFC = 1).

(B) UMAP depiction of cell cycle classification for the scRNA-seq bulk culture landscape.

(C) UMAP representation of scRNA-seq profiles of 28 day bulk cultures, highlighting Leiden clustering (left) and Louvain trajectory inference (right).

(D) UMAP projection of mouse LK/LSK scRNA-seq data (Dahlin *et al*, 2018). Visualization of clusters as identified by the Leiden algorithm informed the selection of the point of origin for DoT score computation.

(E) Gene Set Enrichment Analysis (GSEA) of previously defined hematopoietic cell types (Chambers *et al.*, 2007) using genes upregulated in PosNonELSK cells and NegNonELSK cells.

Appendix Figure S2: RepopSig genes accurately classify different populations of hematopoietic cells.

(A, B) Mean gene expression of MolO (A) and RepopSig genes (B) within each annotated hematopoietic population. The fraction of cells in each group and the mean expression per group are depicted.

Appendix Figure S3: RepopSig genes show high enrichment in single mouse HSCs.

(A) RepopSig gene scoring for hematopoietic cell types, identified by single-cell profiling in Dahlin *et al.,* 2018.

(B) Mean gene expression of RepopSig genes in the scRNA-seq landscape, generated by Dahlin *et al.,* 2018.

Appendix Figure S1

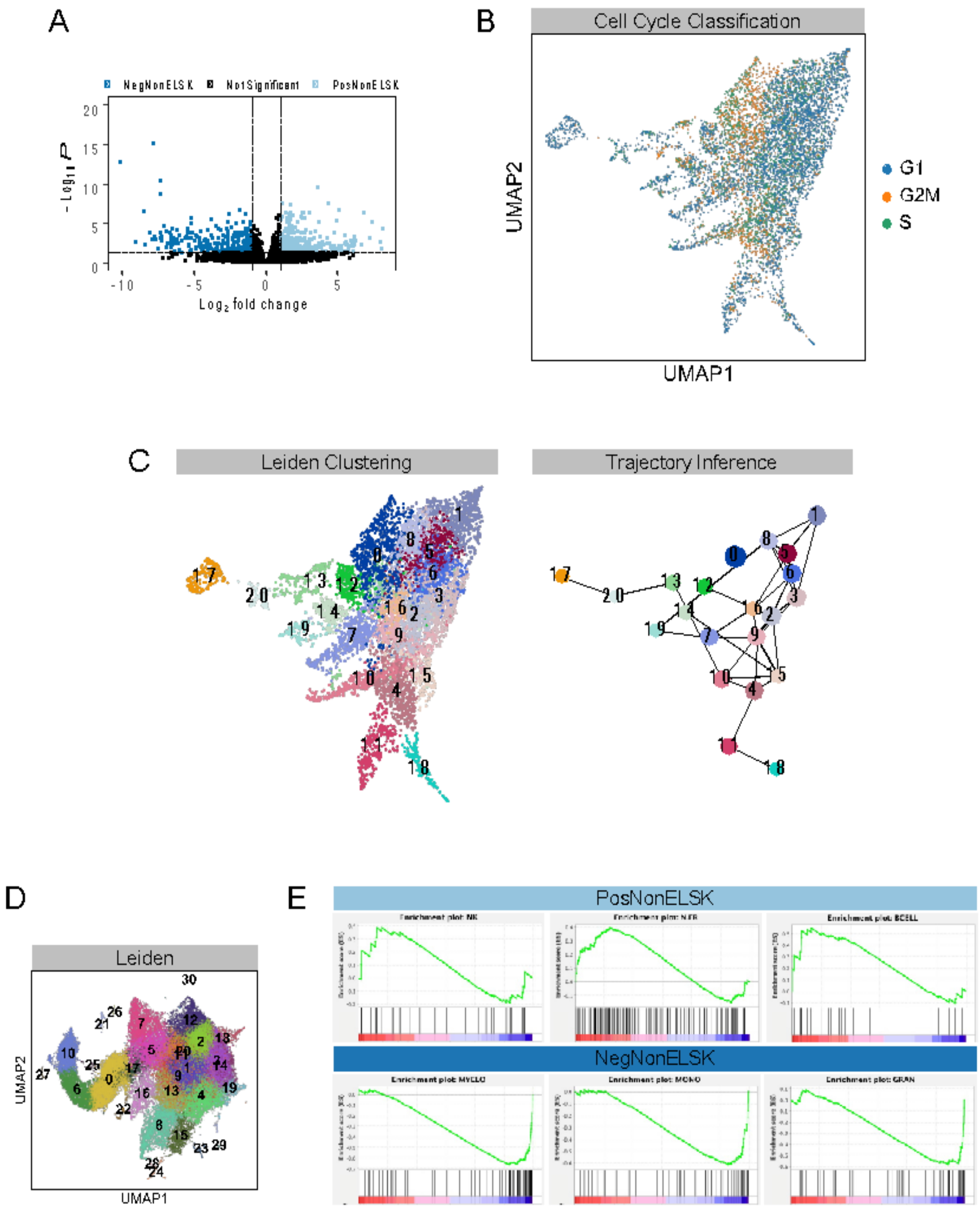

Appendix Figure S2

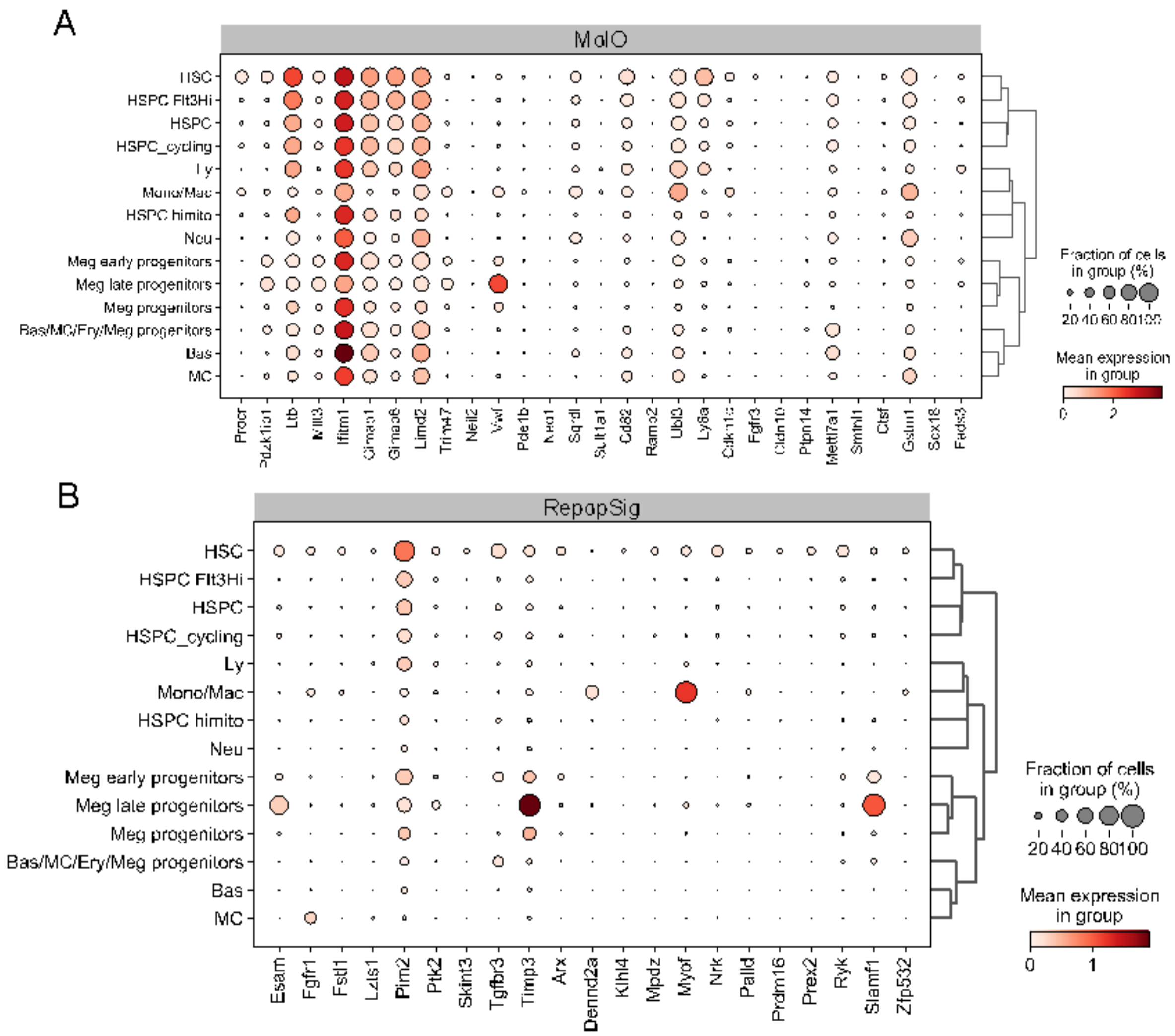

Appendix Figure S3

A

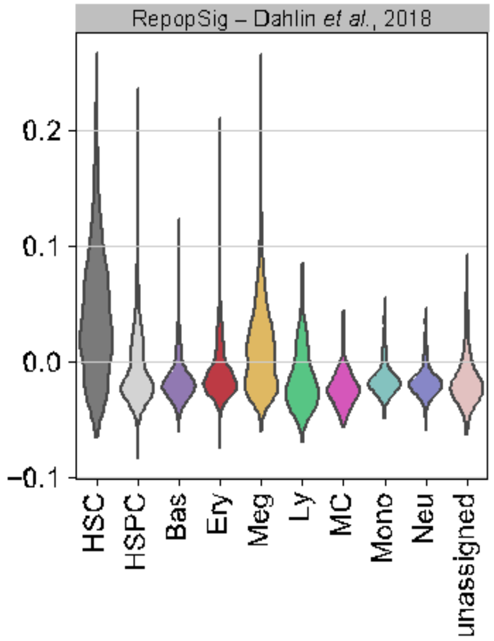

B

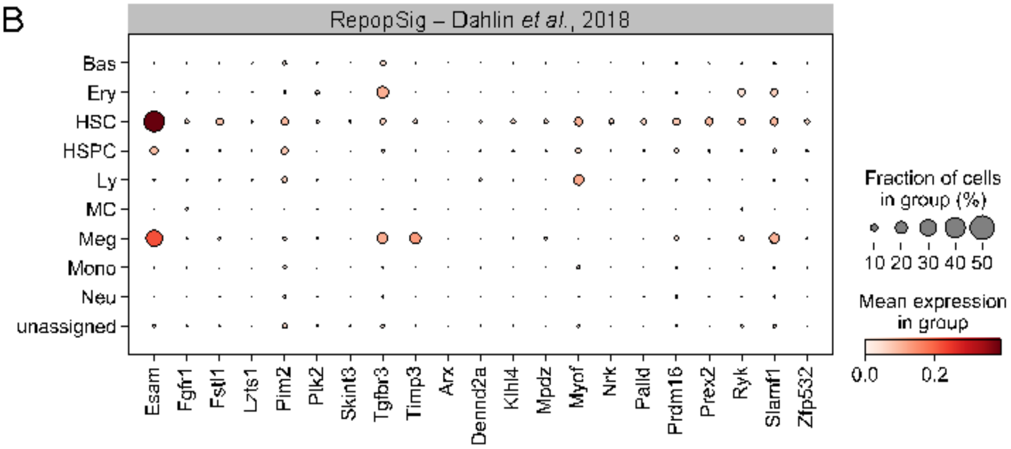

Supplement: Supplementary file 1 — Appendix [file EMBR-23-e55502-s002.pdf]
