# Supplementary material for: Identification and characterization of in vitro expanded hematopoietic stem cells
Source: EMBO Rep. 2022 Aug 16;23(10):e55502. doi: 10.15252/embr.202255502 (PMC9535767; doi:10.15252/embr.202255502)
Supplement: Supplementary file 2 — Expanded View Figures PDF [file EMBR-23-e55502-s003.pdf]

Expanded View Figures

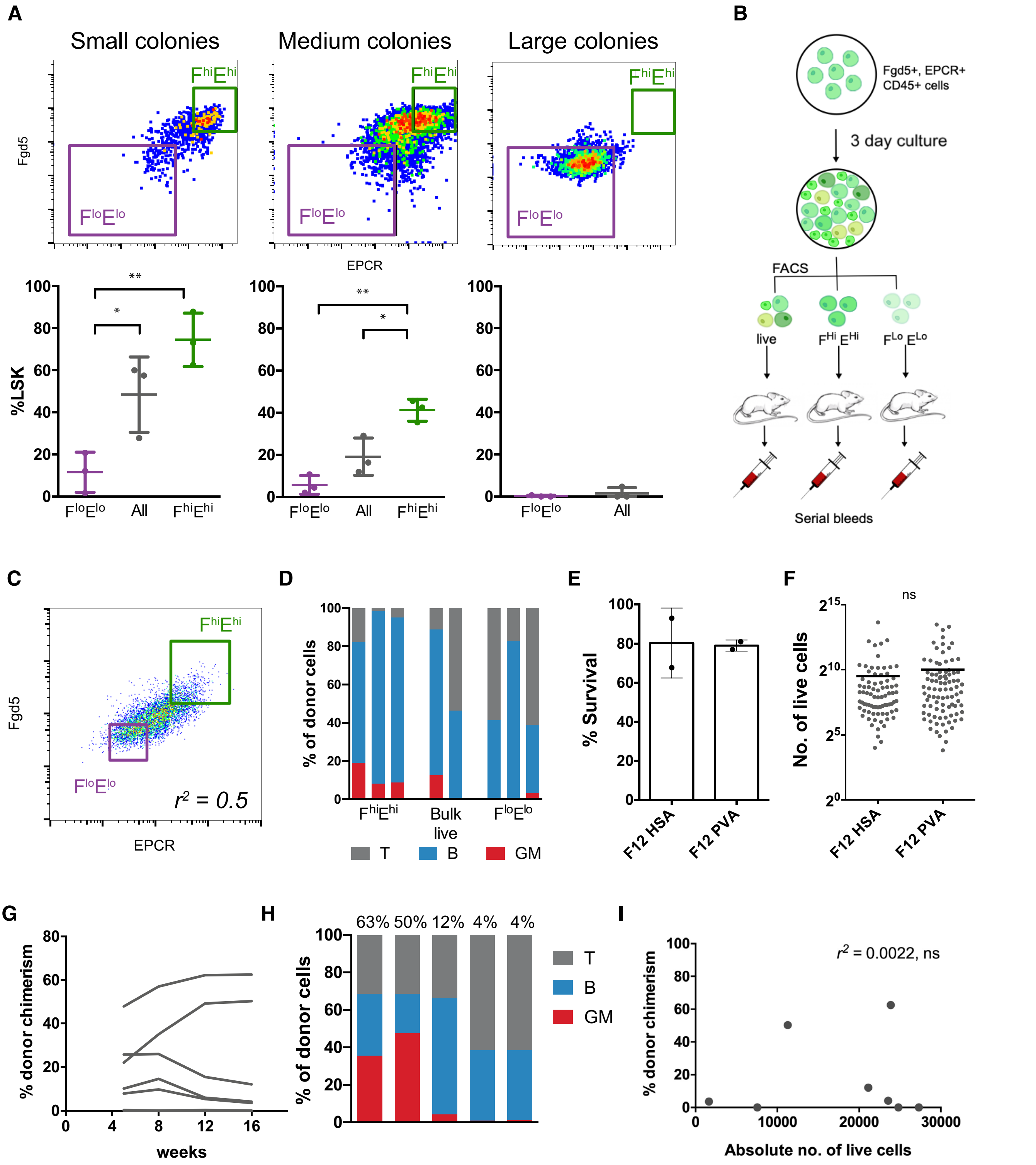

Figure EV1.

 © 2022 The Authors

Figure EV1. All HSC activity is contained within the $F^{hi}E^{hi}$ fraction following culture.

A Representative gating strategy for small (left), medium (middle), large (right) colonies, and the respective LSK percentages within the *Fgd5*^{low}EPCR^{low} and *Fgd5*^{high}EPCR^{high} gates below (n = 3 individual clones per condition). One-way ANOVA, $**P < 0.01$, $*P < 0.05$. Error bars represent SD.
B Schematic of experimental design. *Fgd5* and EPCR^{+} cells were sorted and cultured for 3 days in Stemspan supplemented with 300 ng/ml SCF and 20 ng/ml IL-11, then resorted for *Fgd5*^{high} and EPCR^{high} ($F^{hi}E^{hi}$) and *Fgd5*^{low} and EPCR^{low} ($F^{lo}E^{lo}$) cells for transplantation.
C Gating strategy for $F^{hi}E^{hi}$ and $F^{lo}E^{lo}$ cells.
D Lineage outputs of donor cells from Fig 1D as a percentage of donor cells at 16 weeks post transplantation.
E Clonal survival rates at day 10 in HSA and PVA cultures from Fig 1E (n = 2 biological replicates). Error bars represent SD.
F Clone sizes at day 10 in HSA and PVA cultures.
G Donor chimerism over time for mice transplanted with single clones cultured for 28 days in F12 PVA supplemented with 10 ng/ml SCF, 100 ng/ml TPO, and 20 ng/ml IL-11.
H Lineage output of transplanted clones from (G), as a percentage of donor cells at week 16 post-transplantation. The donor chimerism percentage is labeled above each recipient with above > 1% donor chimerism.
I The relationship between clone size and donor chimerism in transplanted clones from (G). Pearson correlation, ns = not significant.

Figure EV2. The ELSK gating strategy marks repopulating HSCs following culture.

A Representative gating strategy for resort of ELSK and nonELSK cells.
B Donor chimerism and contribution to GM correlated against various phenotypic gating strategies and absolute numbers. Pearson correlation, $****P < 0.0001$, ns = not significant.
C nonELSK cells from Fig 2B were pooled into three separate groups and transplanted into three recipients. Graph shows donor chimerism of the pooled cells and the clones (ELSK cells, 45/50% dose) that they were derived from. On average, each mouse received 26-fold more cells than mice transplanted with ELSK cells.
D Corresponding proportion of cells that were GM, B, and T cell lineages from donor cells out of the three groups at week 16. Donor chimerism is indicated above the bar.
E Number of pooled ELSK cells transplanted for each group.
F Donor chimerism for mice transplanted with single ELSK cells isolated from 28-day cultures initiated from 100 cells, 12-weeks post transplantation. Dotted line indicates 1% chimerism.
G–I Respective GM, B, and T cell lineage contributions of donor single cells at week 12.Dotted lines mark 1% chimerism.
J Donor chimerism from 5% doses of ELSK cells from selected clones from Fig 2B (n = 7, one mouse was culled for health reasons before final timepoint).
K Corresponding lineage output of 5% doses at week 16 as a percentage of donor cells.
L Correlation between donor chimerism and percentage of ELSK in clones that were transplanted at 5% doses. Pearson correlation, $*P < 0.05$.

© 2022 The Authors

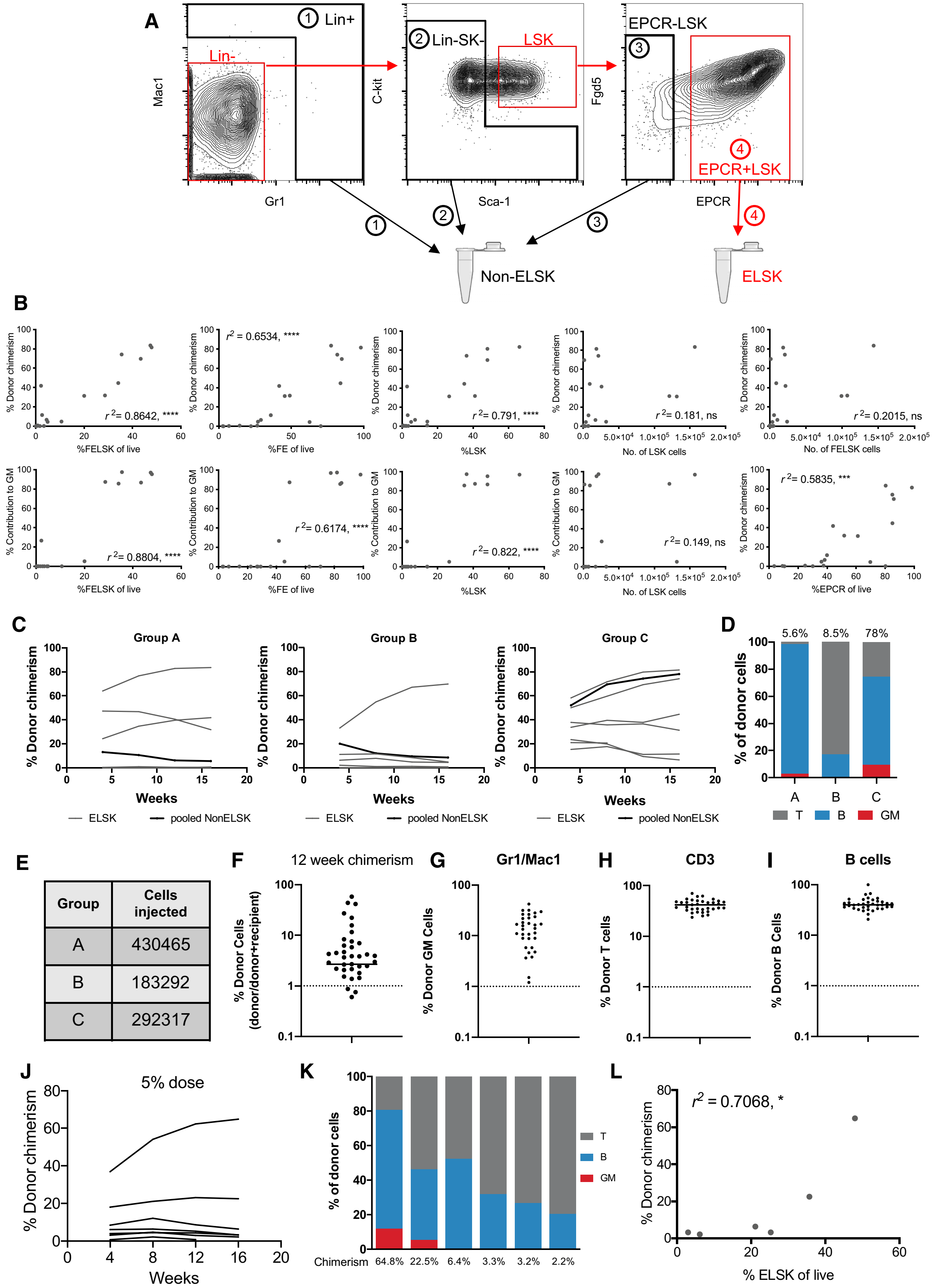

Figure EV2.

 © 2022 The Authors

Figure EV3. Combined RNA-Seq and functional analysis of ELSK and non-ELSK cells following *ex vivo* expansion.

A MDS plots of uncorrected and batch-corrected samples, using category plts (left) and unique sample IDs (right).
B MDS plot of PosELSK and NegELSK samples, colored by donor chimerism and donor GM contribution.
C MolO score of each cell category (geometric mean). Central band represents median, boxes represent first and second quartile, whiskers represent third and fourth quartile and dots represent outliers. *T*-test. Individual geometric means were computed for samples within each defined category.
D Differential gene expression (DGE) plot of PosELSK against NegELSK (P = 0.05 and logFC = 1).
E Heatmap outlining gene expression profiles across NegELSK and PosELSK samples for identified differentially expressed genes (DEGs). N and R numbers on top refer to the specific clones outlined in Table 1.
F GO terms enriched in PosELSK and NegELSK fractions. Computed using differentially expressed genes of repopulating and nonrepopulating ELSK cells.

Source data are available online for this figure.

© 2022 The Authors

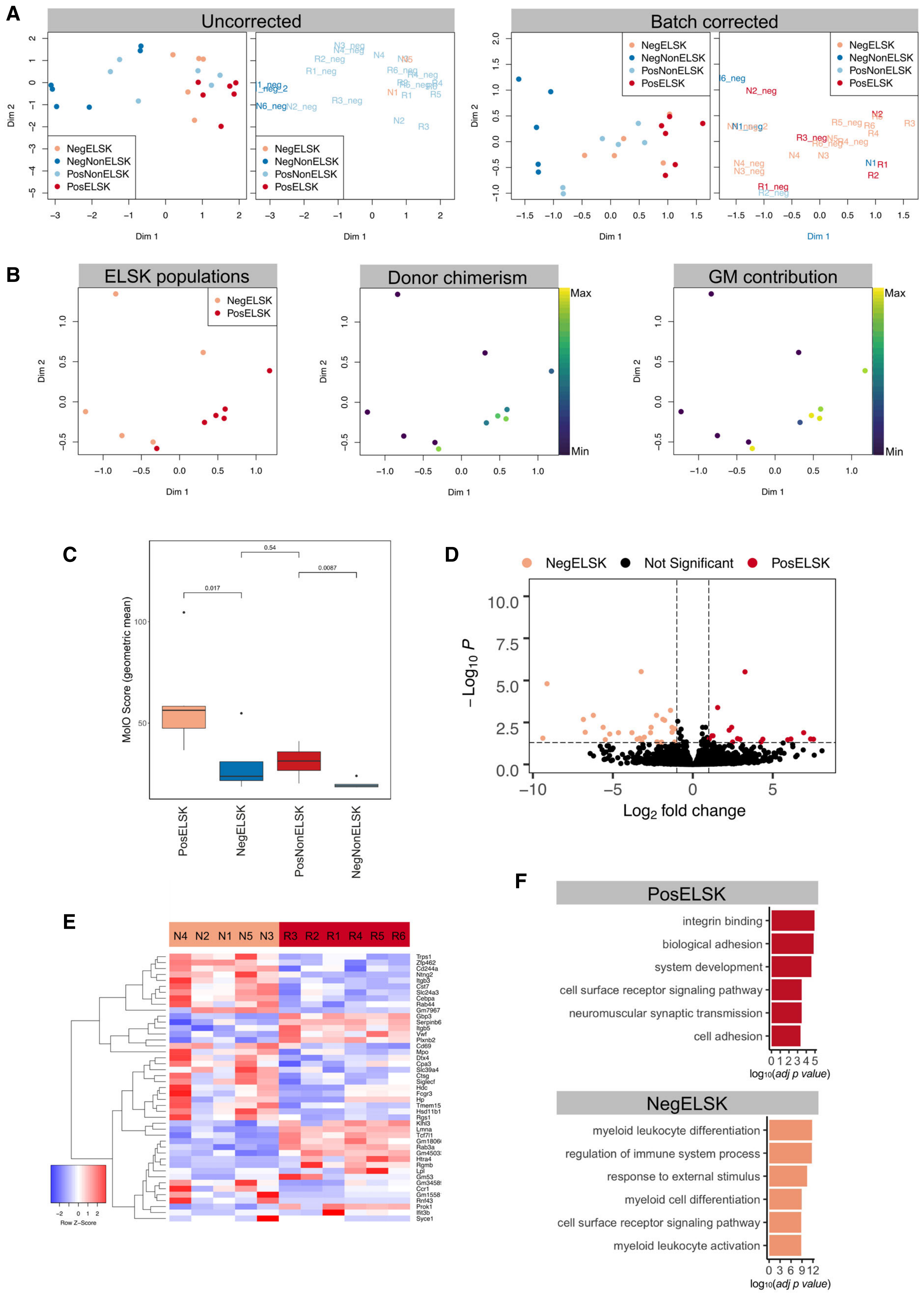

Figure EV3.

 © 2022 The Authors

Figure EV4. Correlation between functional and molecular parameters in cultured HSCs.

A Correlation between the top 7 principal components and the metadata, showing Pearson *r* values and significance. $*P < 0.05$, $**P < 0.01$, $***P < 0.001$, $****P < 0.0001$.

B, C Linear regression plots of individual Repopulation Signature (RepopSig) genes. *P*-value and r^2 value provided for each fitted model.

D RepopSig score representation across the four sample categories Central band represents median, boxes represent first and second quartile, whiskers represent third and fourth quartile and dots represent outliers. *T*-test. Individual geometric means were computed for samples within each defined category.

E MDS plot of all RNA-seq samples, indicating the corresponding MolO score.

F Rank plot depicting the Pearson correlation of each gene with the RepopSig (order by *r* values). MolO signature genes marked in red.

G, H ELSK percentage and live cell numbers of 28-day bulk cultures starting with 50 input cells, with or without titrating doses of CASIN (2, 10, 20 μM), NSC237D66 (5, 50, 200 μM), Rhosin (1, 10, 50 μM) and ML099 (1, 10, 50 μM). $N = 3$–4 individual clones per condition. At all doses tested, CASIN were detrimental to HSC viability. One-way ANOVA. $****P < 0.0001$, $***P < 0.001$, $*P < 0.05$. Error bars represent SD.

© 2022 The Authors

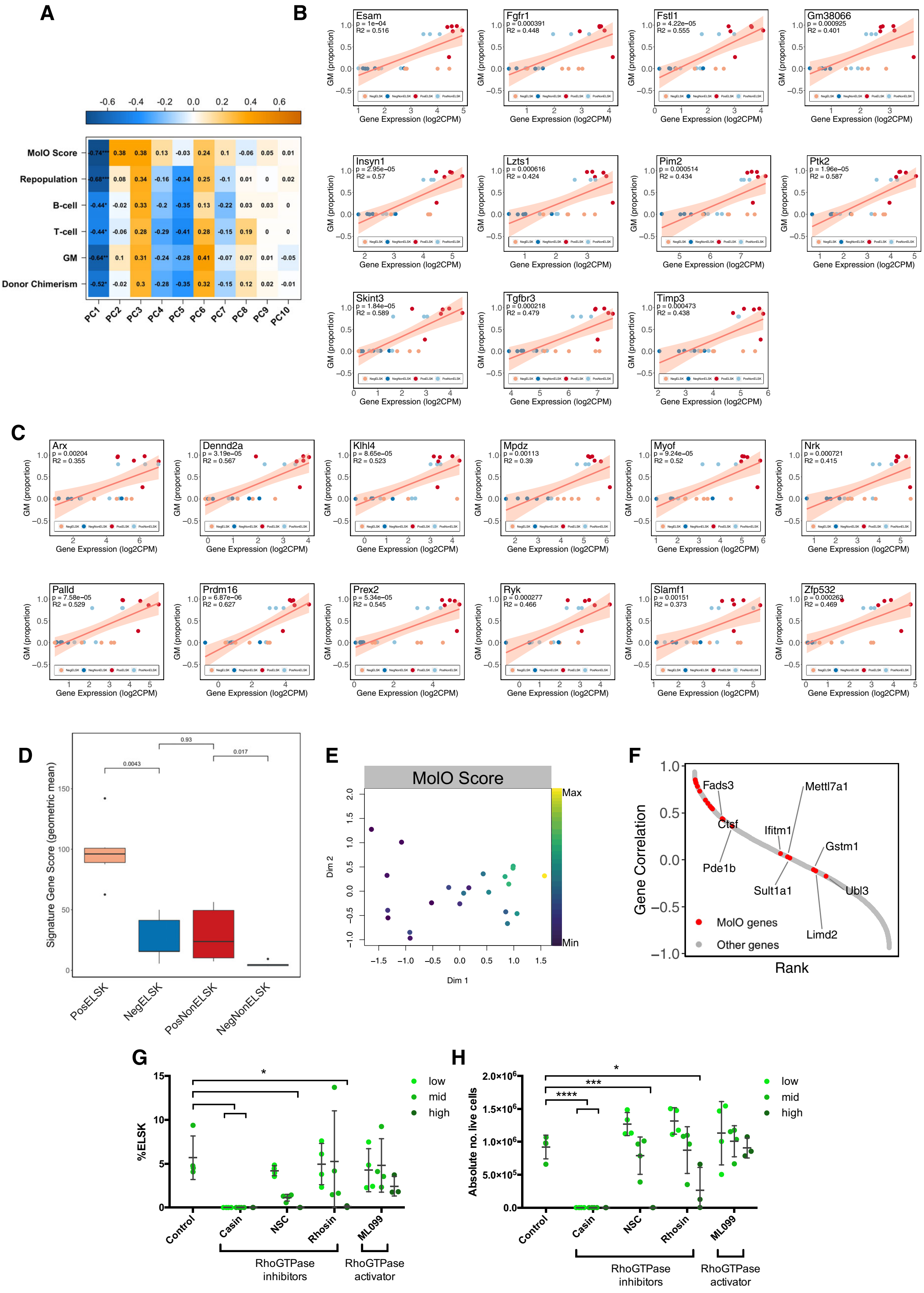

Figure EV4.

 © 2022 The Authors

Figure EV5. RepopSig identifies HSCs across multiple cellular states.

A UMAP representation of scRNA-seq profiles of mouse hematopoietic cells (Nestorowa *et al*, 2016) depicting MolO and RepopSig scores.
B Gene expression profiles of single RepopSig genes.
C UMAP representation of scRNA-seq profiles of freshly isolated HSCs, hibernating HSCs and SCF-stimulated states (Oedekoven *et al*, 2021) showing MolO and RepopSig scores.

© 2022 The Authors

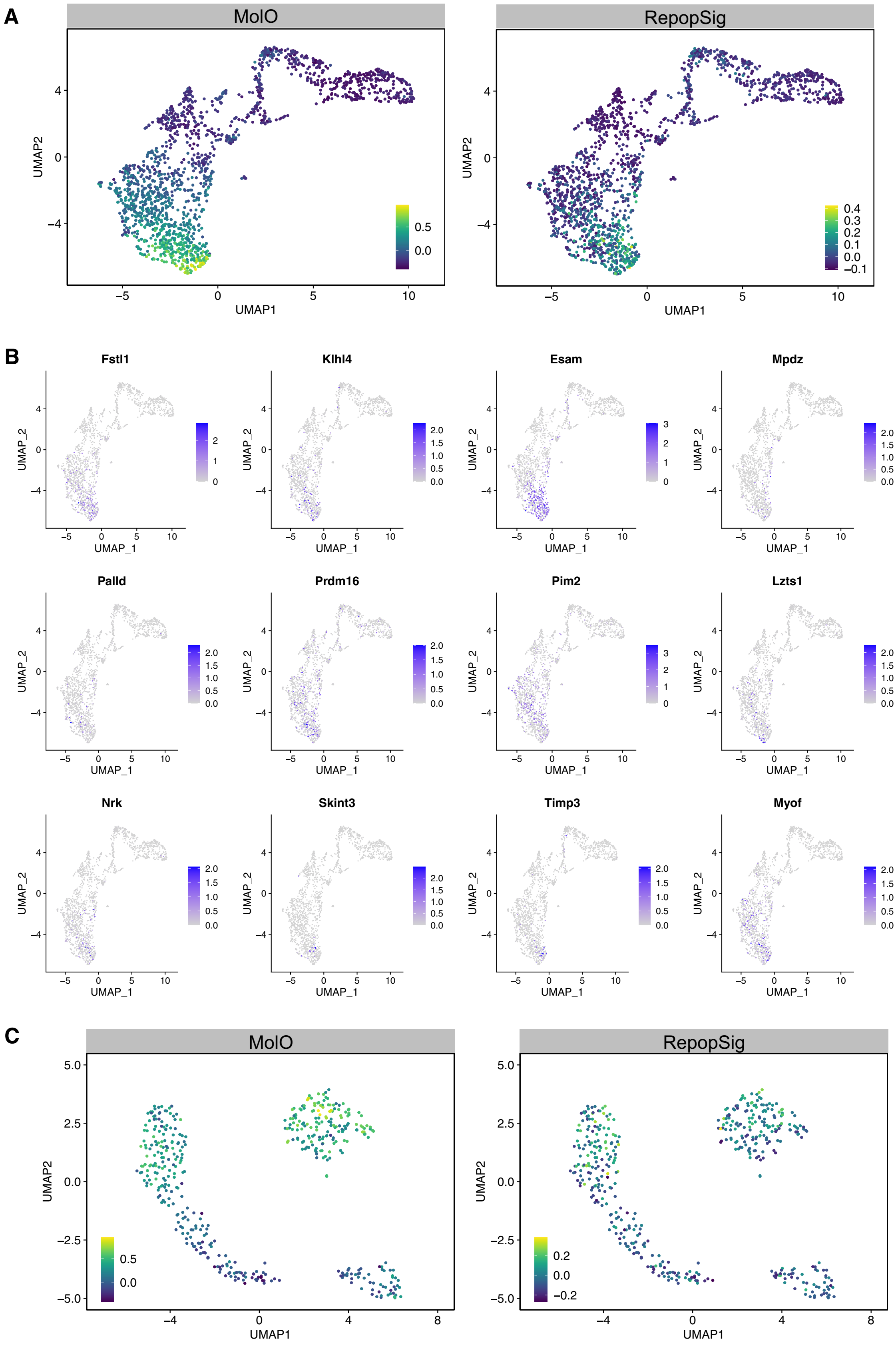

Figure EV5.

 © 2022 The Authors
