# Supplementary figures and images for: Identification and characterization of in vitro expanded hematopoietic stem cells
Source: EMBO Rep. 2022 Aug 16;23(10):e55502. doi: 10.15252/embr.202255502 (PMC9535767; doi:10.15252/embr.202255502)

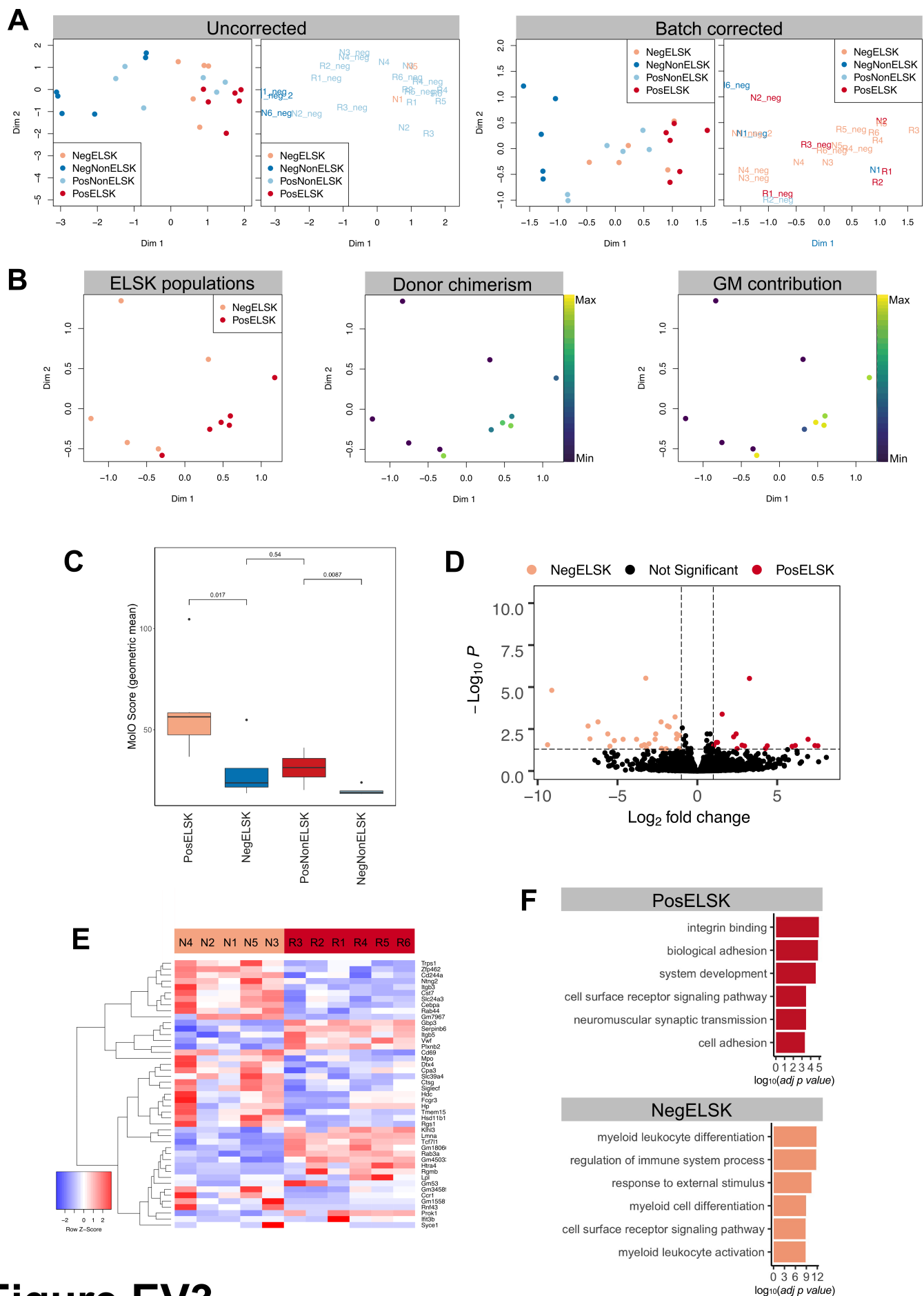

Supplement: Supplementary file 3 — Source Data for Expanded View [file EMBR-23-e55502-s001.pdf]
