# Supplementary material for: Identification and characterization of in vitro expanded hematopoietic stem cells
Source: EMBO Rep. 2022 Aug 16;23(10):e55502. doi: 10.15252/embr.202255502 (PMC9535767; doi:10.15252/embr.202255502)
Supplement: Supplementary file 5 [file EMBR-23-e55502-s004.pdf]

EMBO reports

Identification and characterization of in vitro expanded hematopoietic stem cells

James Che, Daniel Bode, Iwo Kucinski, Alyssa Cull, Fiona Bain, Hans Becker, Maria Jassinskaja, Melania Barile, Grace Boyd, Miriam Belmonte, Andy Zeng, Kyomi Igarashi, Juan Rubio-Lara, Mairi Shepherd, Anna Clay, John Dick, Adam Wilkinson, Hiromitsu Nakauchi, Satoshi Yamazaki, Berthold Gottgens, and David Kent
DOI: 10.15252/embr.202255502

Corresponding author(s): David Kent (david.kent@york.ac.uk)

Review Timeline:

Submission Date:	27th May 22
Editorial Decision:	13th Jun 22
Revision Received:	24th Jun 22
Editorial Decision:	12th Jul 22
Revision Received:	21st Jul 22
Accepted:	27th Jul 22

Editor: Achim Breiling

Transaction Report: Please note that the manuscript was previously reviewed at another journal and the reports were taken into account in the decision-making process at EMBO reports. Since the original reviews are outside of the EMBO Press transparent review process policy, the reports and author response cannot be published.

1st Editorial Decision 13th Jun 2022

Dear Dr. Kent,

Thank you for the submission of your research manuscript to EMBO reports, accompanied with referee reports from a submission to a journal outside EMBO press, and your detailed point-by-point response. I have now received the report from the expert advisor I had contacted to evaluate your revised manuscript, which can be found at the end of this email.

As you will see, the advisor thinks that your manuscript is of interest. However, s/he has three points that should be addressed in a further revised manuscript. Given these constructive comments, I would like to invite you to revise your manuscript with the understanding that these concerns must be addressed in the revised manuscript and in a detailed point-by-point response. Acceptance of your manuscript will depend on a positive outcome of a second assessment of the arbitrator.

Moreover, the manuscript needs formatting according to our journal style. Please carefully review the instructions that follow below.

PLEASE NOTE THAT upon resubmission revised manuscripts are subjected to an initial quality control prior to exposition to re-review. Upon failure in the initial quality control, the manuscripts are sent back to the authors, which may lead to delays. Frequent reasons for such a failure are the lack of the data availability section (please see below) and the presence of statistics based on n=2 (the authors are then asked to present scatter plots or provide more data points).

When submitting your revised manuscript, we will require:

1) a .docx formatted version of the final manuscript text (including legends for main figures, EV figures and tables), but without the figures included. Figure legends should be compiled at the end of the manuscript text.

2) individual production quality figure files as .eps, .tif, .jpg (one file per figure), of main figures and EV figures. Please upload these as separate, individual files upon re-submission.

The Expanded View format, which will be displayed in the main HTML of the paper in a collapsible format, has replaced the Supplementary information. You can submit up to 5 images as Expanded View. Please follow the nomenclature Figure EV1, Figure EV2 etc. The figure legend for these should be included in the main manuscript document file in a section called Expanded View Figure Legends after the main Figure Legends section. Additional Supplementary material should be supplied as a single pdf file labeled Appendix. The Appendix should have page numbers and needs to include a table of content on the first page (with page numbers) and legends for all content. Please follow the nomenclature Appendix Figure Sx, Appendix Table Sx etc. throughout the text, and also label the figures and tables according to this nomenclature.

For more details, please refer to our guide to authors:
http://www.embopress.org/page/journal/14693178/authorguide#manuscriptpreparation

Please consult our guide for figure preparation:
http://wol-prod-cdn.literatumonline.com/pb-assets/embo-site/EMBOPress_Figure_Guidelines_061115-1561436025777.pdf

See also the guidelines for figure legend preparation:
https://www.embopress.org/page/journal/14693178/authorguide#figureformat

3) a complete author checklist, which you can download from our author guidelines (https://www.embopress.org/page/journal/14693178/authorguide). Please insert page numbers in the checklist to indicate where the requested information can be found in the manuscript. The completed author checklist will also be part of the RPF.

Please also follow our guidelines for the use of living organisms, and the respective reporting guidelines:
http://www.embopress.org/page/journal/14693178/authorguide#livingorganisms

4) that primary datasets produced in this study (e.g. RNA-seq, ChIP-seq, structural and array data) are deposited in an appropriate public database. If no primary datasets have been deposited, please also state this in a dedicated section (e.g. 'No primary datasets have been generated and deposited'), see below.

See also: http://embor.embopress.org/authorguide#datadeposition

Please remember to provide a reviewer password if the datasets are not yet public.

The accession numbers and database should be listed in a formal "Data Availability" section (placed after Materials & Methods) that follows the model below. This is now mandatory (like the COI statement). Please note that the Data Availability Section is restricted to new primary data that are part of this study. This section is mandatory. As indicated above, if no primary datasets

have been deposited, please state this in this section

Data availability

The datasets produced in this study are available in the following databases:

- RNA-Seq data: Gene Expression Omnibus GSE46843 (https://www.ncbi.nlm.nih.gov/geo/query/acc.cgi?acc=GSE46843)
- [data type]: [name of the resource] [accession number/identifier/doi] ([URL or identifiers.org/DATABASE:ACCESSION])

*** Note - All links should resolve to a page where the data can be accessed. ***

Moreover, I have these editorial requests:

6) We strongly encourage the publication of original source data with the aim of making primary data more accessible and transparent to the reader. The source data will be published in a separate source data file online along with the accepted manuscript and will be linked to the relevant figure. If you would like to use this opportunity, please submit the source data (for example scans of entire gels or blots, data points of graphs in an excel sheet, additional images, etc.) of your key experiments together with the revised manuscript. If you want to provide source data, please include size markers for scans of entire gels, label the scans with figure and panel number, and send one PDF file per figure.

7) Our journal encourages inclusion of *data citations in the reference list* to directly cite datasets that were re-used and obtained from public databases. Data citations in the article text are distinct from normal bibliographical citations and should directly link to the database records from which the data can be accessed. In the main text, data citations are formatted as follows: "Data ref: Smith et al, 2001" or "Data ref: NCBI Sequence Read Archive PRJNA342805, 2017". In the Reference list, data citations must be labeled with "[DATASET]". A data reference must provide the database name, accession number/identifiers and a resolvable link to the landing page from which the data can be accessed at the end of the reference. Further instructions are available at: http://www.embopress.org/page/journal/14693178/authorguide#referencesformat

8) Regarding data quantification and statistics, please make sure that the number "n" for how many independent experiments were performed, their nature (biological versus technical replicates), the bars and error bars (e.g. SEM, SD) and the test used to calculate p-values is indicated in the respective figure legends (also for potential EV figures and all those in the final Appendix). Please also check that all the p-values are explained in the legend, and that these fit to those shown in the figure. Please provide statistical testing where applicable. Please avoid the phrase 'independent experiment', but clearly state if these were biological or technical replicates. See also:
http://www.embopress.org/page/journal/14693178/authorguide#statisticalanalysis

9) Please also note our reference format:
http://www.embopress.org/page/journal/14693178/authorguide#referencesformat

10) We updated our journal's competing interests policy in January 2022 and request authors to consider both actual and perceived competing interests. Please review the policy https://www.embopress.org/competing-interests and update your competing interests if necessary. Please name this section 'Disclosure and Competing Interests Statement' and put it after the Acknowledgements section.

11) Please remove the key points from the title page (these can be used for the bullet points - see below), change the name of the 'Summary ' into 'Abstract', put up to 5 key words to the title page, and order the manuscript sections like this (using these names):
Title page - Abstract - Key words - Introduction - Results - Discussion - Materials and Methods - Data Availability Section (DAS) - Acknowledgements - Author contributions - Disclosure and Competing Interests Statement - References - Figure legends - Expanded View Figure legends

12) Please include all the methods information in the main manuscript text.

In addition, I would need from you (please upload these a separate related manuscript files):
- a short, two-sentence summary of the manuscript (not more than 35 words).
- three to four short bullet points highlighting the key findings of your study.
- a schematic summary figure (synopsis image) in jpeg or tiff format with the exact width of 550 pixels and a height of not more than 400 pixels that can be used as a visual synopsis on our website.

I look forward to seeing a revised version of your manuscript when it is ready. Please let me know if you have questions or comments regarding the revision.

Please use this link to submit your revision: https://embor.msubmit.net/cgi-bin/main.plex

Yours sincerely,

Achim Breiling
Senior Editor
EMBO Reports

Arbitrator:

As noted by Che et al., an important limitation in the field of HSC biology is the inability to identify HSCs in culture with existing markers. Markers that work well to identify HSCs in vivo do not work in culture because cultured HSCs are scattered among positive and negative fractions for all of the markers that are most informative in vivo. Che et al. argue that they have succeeded in prospectively identifying HSCs from cultured clones. This implies that their markers give a high level of HSC purity in the positive fraction (EPCR+ESAM+) and few or no HSCs in the negative fraction (all other cells from the same cultures). They provide a lot of correlational data that indicate that cultured HSCs are enriched in the EPCR+ESAM+ fraction but, disappointingly, they never directly test the purity of HSCs in either the positive or negative fractions. Without a more quantitative analysis of HSC purity, it's not clear how much better these markers are as compared to other markers. The authors should pick their best markers and do a single cell or limiting dilution analysis to determine HSC purity in transplantation assays for the positive and negative fractions.

1. What percentage of single cells, identified with the authors best markers (since the authors use different combinations of markers in different experiments it's not entirely clear to me what the best combination is), give HSC activity upon competitive transplantation into irradiated mice? If few single cells would reconstitute, the authors could transplant small numbers of cells (5 or 10?) so as to perform a limit dilution analysis of HSC frequency. Without doing that, we have no information about HSC purity in the population identified by the authors. Without information on HSC purity, it's hard to interpret the gene expression profile or the extent to which it reflects highly purified HSCs.
2. The converse question, is what percentage of HSCs in these cultures fall outside of the positive fraction. The authors did perform transplantation assays in which they show that HSC activity is depleted in the negative fraction as compared to the positive fraction. However, these assays weren't done in a quantitative way (it wasn't clear to me how many cells were transplanted in many experiments), or at limit dilution. Consequently, it isn't clear what percentage of HSCs fall outside of the positive fraction, or to what extent HSCs in the negative fraction are depleted as compared to unfractionated cells from the same cultures. Without this information, we just don't know how good the authors' markers are at prospectively identifying HSCs in culture.
3. If the authors could show that their markers give high levels of purity for cultured mouse HSCs and that few HSCs are present in the negative fraction, this would be an important advance for the field irrespective of whether the same markers work to identify human HSCs. Until such information is available, it seems premature to rely on gene expression signatures from these cells.

Thank you for sending our paper out for an arbitrating reviewer's assessment. We have now addressed both of the core concerns and hope that these changes and new datasets will make our paper acceptable for publication in EMBO Reports.

For ease, I have included the arbitrating reviewer's comments in black and our responses in blue.

----

1. What percentage of single cells, identified with the authors best markers (since the authors use different combinations of markers in different experiments it's not entirely clear to me what the best combination is), give HSC activity upon competitive transplantation into irradiated mice? If few single cells would reconstitute, the authors could transplant small numbers of cells (5 or 10?) so as to perform a limit dilution analysis of HSC frequency. Without doing that, we have no information about HSC purity in the population identified by the authors. Without information on HSC purity, it's hard to interpret the gene expression profile or the extent to which it reflects highly purified HSCs.

We have now provided new single cell transplantation data in the manuscript (Supplemental Figure 2F - 2I). At 12 weeks post-transplantation, 33 of 50 (66%) single cell transplantations gave donor chimerism values of >1% (Figure S2F). Within this, 17 of 50 mice (34%) contributed >0.5% to each of the GM, B and T cell lineages (Figure S2G-I). Importantly, a number of these single ELSK cell repopulated (10 mice, 20% of the total) mice have donor cell contribution of more than 20% of the total blood cells.

2. The converse question, is what percentage of HSCs in these cultures fall outside of the positive fraction. The authors did perform transplantation assays in which they show that HSC activity is depleted in the negative fraction as compared to the positive fraction. However, these assays weren't done in a quantitative way (it wasn't clear to me how many cells were transplanted in many experiments), or at limit dilution. Consequently, it isn't clear what percentage of HSCs fall outside of the positive fraction, or to what extent HSCs in the negative fraction are depleted as compared to unfractionated cells from the same cultures. Without this information, we just don't know how good the authors' markers are at prospectively identifying HSCs in culture.

We now provide a calculation of HSC frequency of the non-ELSK fraction from our transplantation data. These experiments assessed 50% of the total non-ELSK cells from 19 different clones and we can estimate the HSC frequency as less than 1 in 750,494 based on the transplantation data. We believe this represents very strong evidence that the negative fraction has very few HSCs and apologise that this was not presented clearly in the paper. We have included the following text in the revised manuscript:

"…nonELSK cells largely lacked multilineage reconstitution capacity (Figure S2C-D) with a calculated estimate of HSC frequency within the nonELSK fraction being less than 1 in 750,494 cells (Figure S2E), strongly indicating that the vast majority of functional HSCs are contained within the ELSK fraction."

3. If the authors could show that their markers give high levels of purity for cultured mouse HSCs and that few HSCs are present in the negative fraction, this would be an important

advance for the field irrespective of whether the same markers work to identify human HSCs. Until such information is available, it seems premature to rely on gene expression signatures from these cells.

We are confident that the answers to points 1 and 2 address these concerns with the new single cell transplantation indicating a very high functional purity of HSCs within the ELSK fraction.

1st Revision - Editorial Decision 12th Jul 2022

Dear Dr. Kent

Thank you for the submission of your further revised manuscript to our editorial offices. I have now received the report from the arbitrator that was asked to evaluate your study, you will find below. As you will see, the arbitrator now supports the publication of the study in EMBO reports. The arbitrator has a final request/suggestion to improve the manuscript I ask you to address in a final revised manuscript. Please also provide a final p-b-p-response addressing the remaining point of the arbitrator.

Moreover, I have these editorial requests:

- Please provide a title with not more than 100 characters (including spaces).

- Please add up to 5 keywords to the title page.

- Please remove the key points from the manuscript text file. These can be used for the bullet points (see below).

- Please name the 'Summary' 'Abstract' and make sure this has not more than 175 words.

- Please provide individual production quality figure files as .eps, .tif, .jpg (one file per figure), of main figures and EV figures. Please upload these as separate, individual files upon re-submission.

The Expanded View format, which will be displayed in the main HTML of the paper in a collapsible format, has replaced the Supplementary information. You can submit up to 5 images as Expanded View. Please follow the nomenclature Figure EV1, Figure EV2 etc. The figure legend for these should be included in the main manuscript document file in a section called Expanded View Figure Legends after the main Figure Legends section. Additional Supplementary material should be supplied as a single pdf file labeled Appendix. The Appendix should have page numbers and needs to include a table of content on the first page (with page numbers) and legends for all content. Please follow the nomenclature Appendix Figure Sx, Appendix Table Sx etc. throughout the text, and also label the figures and tables according to this nomenclature.

For more details, please refer to our guide to authors:
http://www.embopress.org/page/journal/14693178/authorguide#manuscriptpreparation

Please consult our guide for figure preparation:
http://wol-prod-cdn.literatumonline.com/pb-assets/embo-site/EMBOPress_Figure_Guidelines_061115-1561436025777.pdf

See also the guidelines for figure legend preparation:
https://www.embopress.org/page/journal/14693178/authorguide#figureformat

- We updated our journal's competing interests policy in January 2022 and request authors to consider both actual and perceived competing interests. Please review the policy https://www.embopress.org/competing-interests and update your competing interests if necessary. Please name this section 'Disclosure and Competing Interests Statement' and put it after the Acknowledgements section.

- Please deposit the primary datasets produced in this study (e.g. RNA-seq, ChIP-seq, structural and array data) in an appropriate public database.

See also: http://embor.embopress.org/authorguide#datadeposition

Please make sure that the datasets are public latest upon publication of the study.

The accession numbers and database should be listed in a formal "Data Availability" section (placed after Materials & Methods) that follows the model below. This is now mandatory (like the COI statement). Please note that the Data Availability Section is restricted to new primary data that are part of this study. This section is mandatory. As indicated above, if no primary datasets have been deposited, please state this in this section

Data availability

The datasets produced in this study are available in the following databases:

- RNA-Seq data: Gene Expression Omnibus GSE46843 (https://www.ncbi.nlm.nih.gov/geo/query/acc.cgi?acc=GSE46843)
- [data type]: [name of the resource] [accession number/identifier/doi] ([URL or identifiers.org/DATABASE:ACCESSION])

*** Note - All links should resolve to a page where the data can be accessed. ***

- Please provide all the materials and methods information (and their references) in the main manuscript text. Please remove this from the Appendix file.

- Please order the manuscript sections like this, using these names:
Title page - Abstract - Key Words - Introduction - Results - Discussion - Materials and Methods - Data availability section - Acknowledgements - Author contributions - Disclosure and Competing Interests Statement - References - Figure legends - Expanded View Figure legends

- It seems separate callouts for panels S1I, S2A, S3A are missing. Please check and make sure that all figure panels are called out separately and sequentially.

- Please make sure that the number "n" for how many independent experiments were performed, their nature (biological versus technical replicates), the bars and error bars (e.g. SEM, SD) and the test used to calculate p-values is indicated in the respective figure legends (main, EV and Appendix figures), and that statistical testing has been done where applicable. Please avoid phrases like 'independent experiment', but clearly state if these were biological or technical replicates. Please add complete statistical testing to all diagrams (for main, EV and Appendix figures). Please also indicate (e.g. with n.s.) if testing was performed, but the differences are not significant.

- Finally, please find attached a word file of the manuscript text (provided by our publisher) with changes we ask you to include in your final manuscript text, and some queries, we ask you to address. Please provide your final manuscript file with track changes, in order that we can see any modifications done.

In addition, I would need from you:
- a short, two-sentence summary of the manuscript (around 35 words).
- three to four short bullet points highlighting the key findings of your study
- a schematic summary figure (in jpeg or tiff format with the exact width of 550 pixels and a height of not more than 400 pixels) that can be used as a visual synopsis on our website.

I look forward to seeing the final revised version of your manuscript when it is ready. Please let me know if you have questions regarding the revision.

Please use this link to submit your revision: https://embor.msubmit.net/cgi-bin/main.plex

Best,

Achim Breiling
Senior Editor
EMBO Reports

Arbitrator:

The activity detected by the authors in ELSK transplantation assays is impressive. There is one last piece of information the authors should add to address point #2 from my last set of reviewer comments. Based on the percentage of cells that fall within the ELSK and non-ELSK fractions in their cultures, what percentage of all HSCs in the cultures they studied fall within the ELSK versus non-ELSK fractions? For example, if non-ESLK cells represent 99% of the cells in the cultures and HSCs are 1000-fold depleted in this fraction as compared to the ELSK fraction, then 10% of all HSCs would fall in the non-ELSK fraction. The authors should add this piece of information to the revised manuscript but it would not need re-review.

Dear Achim,

Thank you again for accepting our manuscript for publication in *EMBO Reports*. We have now completed the final checks and submit our updated documents.

In response to the final reviewer comment (below):
"There is one last piece of information the authors should add to address point #2 from my last set of reviewer comments. Based on the percentage of cells that fall within the ELSK and non-ELSK fractions in their cultures, what percentage of all HSCs in the cultures they studied fall within the ELSK versus non-ELSK fractions?… The authors should add this piece of information to the revised manuscript but it would not need re-review."

The arbitrating reviewer has requested that we utilise the frequency of HSCs from the non-HSC fraction (1 in 750,494 cells (Figure EV2E) from pooled non-ELSK cells from sets of clones) and the HSC fraction (~50% based on single ELSK transplantation from bulk cultures) to try and work out the fraction of total HSCs that are not present in the HSC fraction. Since our HSC frequency calculation was from bulk cultures and our non-HSC frequency calculation was from single clones, it is extremely difficult to try and ascribe a number as the experimental setup is different and the resulting estimate would be potentially confounded. We have therefore opted to avoid a formal estimate of frequency which we do not think we can responsibly provide and instead indicate on page 6, lines 177-180 that some functional HSCs are likely to exist outside the ELSK phenotype:

"These data indicate that the ELSK fraction is highly enriched for HSCs (>100,000 fold) compared to non-ELSK cells although a small minority of functional HSCs likely still exist outside of the ELSK gate"

We hope that these changes are acceptable.

Best regards,
David

2nd Revision - Editorial Decision 27th Jul 2022

Dr. David Kent
York Biomedical Research Institute, University of York
Biology
United Kingdom

Dear Dr. Kent,

I am very pleased to accept your manuscript for publication in the next available issue of EMBO reports. Thank you for your contribution to our journal.

At the end of this email I include important information about how to proceed. Please ensure that you take the time to read the information and complete and return the necessary forms to allow us to publish your manuscript as quickly as possible.

As part of the EMBO publication's Transparent Editorial Process, EMBO reports publishes online a Review Process File to accompany accepted manuscripts. As you are aware, this File will be published in conjunction with your paper and will include the referee reports, your point-by-point response and all pertinent correspondence relating to the manuscript.

If you do NOT want this File to be published, please inform the editorial office within 2 days, if you have not done so already, otherwise the File will be published by default [contact: emboreports@embo.org]. If you do opt out, the Review Process File link will point to the following statement: "No Review Process File is available with this article, as the authors have chosen not to make the review process public in this case."

Thank you again for your contribution to EMBO reports and congratulations on a successful publication. Please consider us again in the future for your most exciting work.

Yours sincerely,

Achim Breiling
Senior Editor
EMBO Reports

****

THINGS TO DO NOW:

Please note that you will be contacted by Wiley Author Services to complete licensing and payment information. The required 'Page Charges Authorization Form' is available here: https://www.embopress.org/pb-assets/embo-site/er_apc.pdf - please download and complete the form and return to embopressproduction@wiley.com

You will receive proofs by e-mail approximately 2-3 weeks after all relevant files have been sent to our Production Office; you should return your corrections within 2 days of receiving the proofs.

Please inform us if there is likely to be any difficulty in reaching you at the above address at that time. Failure to meet our deadlines may result in a delay of publication, or publication without your corrections.

All further communications concerning your paper should quote reference number EMBOR-2022-55502V3 and be addressed to emboreports@wiley.com.

Should you be planning a Press Release on your article, please get in contact with emboreports@wiley.com as early as possible, in order to coordinate publication and release dates.

EMBO Press Author Checklist

Corresponding Author Name: David Kent	
Journal Submitted to: EMBO Reports	
Manuscript Number: EMBOR-2022-55502V2	

USEFUL LINKS FOR COMPLETING THIS FORM
The EMBO Journal - Author Guidelines
EMBO Reports - Author Guidelines
Molecular Systems Biology - Author Guidelines
EMBO Molecular Medicine - Author Guidelines

Reporting Checklist for Life Science Articles (updated January 2022)
This checklist is adapted from Materials Design Analysis Reporting (MDAR) Checklist for Authors. MDAR establishes a minimum set of requirements in transparent reporting in the life sciences (see Statement of Task: 10.31222/osf.io/9sm4x). Please follow the journal's guidelines in preparing your manuscript.
Please note that a copy of this checklist will be published alongside your article.

Abridged guidelines for figures
1. Data
The data shown in figures should satisfy the following conditions:
- the data were obtained and processed according to the field's best practice and are presented to reflect the results of the experiments in an accurate and unbiased manner.
- ideally, figure panels should include only measurements that are directly comparable to each other and obtained with the same assay.
- plots include clearly labeled error bars for independent experiments and sample sizes. Unless justified, error bars should not be shown for technical replicates.
- if n<5, the individual data points from each experiment should be plotted. Any statistical test employed should be justified.
- Source Data should be included to report the data underlying figures according to the guidelines set out in the authorship guidelines on Data Presentation.

2. Captions
Each figure caption should contain the following information, for each panel where they are relevant:
- a specification of the experimental system investigated (eg cell line, species name).
- the assay(s) and method(s) used to carry out the reported observations and measurements.
- an explicit mention of the biological and chemical entity(ies) that are being measured.
- an explicit mention of the biological and chemical entity(ies) that are altered/varied/perturbed in a controlled manner.
- the exact sample size (n) for each experimental group/condition, given as a number, not a range;
- a description of the sample collection allowing the reader to understand whether the samples represent technical or biological replicates (including how many animals, litters, cultures, etc.).
- a statement of how many times the experiment shown was independently replicated in the laboratory.
- definitions of statistical methods and measures:
  - common tests, such as t-test (please specify whether paired vs. unpaired), simple χ2 tests, Wilcoxon and Mann-Whitney tests, can be unambiguously identified by name only, but more complex techniques should be described in the methods section;
  - are tests one-sided or two-sided?
  - are there adjustments for multiple comparisons?
  - exact statistical test results, e.g., P values = x but not P values < x;
  - definition of 'center values' as median or average;
  - definition of error bars as s.d. or s.e.m.

Please complete ALL of the questions below.
Select "Not Applicable" only when the requested information is not relevant for your study.

Materials

Newly Created Materials	Information included in the manuscript?	In which section is the information available? (Reagents and Tools Table, Materials and Methods, Figures, Data Availability Section)
New materials and reagents need to be available; do any restrictions apply?	Yes	Methods, Supplemental

Antibodies	Information included in the manuscript?	In which section is the information available? (Reagents and Tools Table, Materials and Methods, Figures, Data Availability Section)
For **antibodies** provide the following information:<br>- Commercial antibodies: RRID (if possible) or supplier name, catalogue number and or/clone number<br>- Non-commercial: RRID or citation	Yes	Methods, Supplemental

DNA and RNA sequences	Information included in the manuscript?	In which section is the information available? (Reagents and Tools Table, Materials and Methods, Figures, Data Availability Section)
**Short novel DNA or RNA including primers, probes**: provide the sequences.	Not Applicable	

Cell materials	Information included in the manuscript?	In which section is the information available? (Reagents and Tools Table, Materials and Methods, Figures, Data Availability Section)
**Cell lines:** Provide species information, strain. Provide accession number in repository **OR** supplier name, catalog number, clone number, and/**OR** RRID.	Not Applicable	
**Primary cultures:** Provide species, strain, sex of origin, genetic modification status.	Yes	Methods, Supplemental
Report if the cell lines were recently **authenticated** (e.g., by STR profiling) and tested for mycoplasma contamination.	Not Applicable	

Experimental animals	Information included in the manuscript?	In which section is the information available? (Reagents and Tools Table, Materials and Methods, Figures, Data Availability Section)
**Laboratory animals or Model organisms:** Provide species, strain, sex, age, genetic modification status. Provide accession number in repository **OR** supplier name, catalog number, clone number, **OR** RRID.	Yes	Methods, Supplemental
**Animal observed in or captured from the field:** Provide species, sex, and age where possible.	Not Applicable	
Please detail **housing and husbandry conditions**.	Yes	Methods, Supplemental

Plants and microbes	Information included in the manuscript?	In which section is the information available? (Reagents and Tools Table, Materials and Methods, Figures, Data Availability Section)
**Plants:** provide species and strain, ecotype and cultivar where relevant, unique accession number if available, and source (including location for collected wild specimens).	Not Applicable	
**Microbes:** provide species and strain, unique accession number if available, and source.	Not Applicable	

Human research participants	Information included in the manuscript?	In which section is the information available? (Reagents and Tools Table, Materials and Methods, Figures, Data Availability Section)
If collected and within the bounds of privacy constraints report on age, sex and gender or ethnicity for all study participants.	Not Applicable	

Core facilities	Information included in the manuscript?	In which section is the information available? (Reagents and Tools Table, Materials and Methods, Figures, Data Availability Section)
If your work benefited from core facilities, was their service mentioned in the acknowledgments section?	Yes	Acknowledgements

Design

Study protocol	Information included in the manuscript?	In which section is the information available? (Reagents and Tools Table, Materials and Methods, Figures, Data Availability Section)
If study protocol has been **pre-registered, provide DOI in the manuscript**. For clinical trials, provide the trial registration number **OR** cite DOI.	Not Applicable	
Report the **clinical trial registration number** (at ClinicalTrials.gov or equivalent), where applicable.	Not Applicable	

Laboratory protocol	Information included in the manuscript?	In which section is the information available? (Reagents and Tools Table, Materials and Methods, Figures, Data Availability Section)
Provide DOI OR other citation details if **external detailed step-by-step protocols** are available.	Not Applicable	

Experimental study design and statistics	Information included in the manuscript?	In which section is the information available? (Reagents and Tools Table, Materials and Methods, Figures, Data Availability Section)
Include a statement about **sample size** estimate even if no statistical methods were used.	Yes	Methods, Supplemental, Figure Legends
Were any steps taken to minimize the effects of subjective bias when allocating animals/samples to treatment (e.g. **randomization procedure**)? If yes, have they been described?	Not Applicable	
Include a statement about **blinding** even if no blinding was done.	Not Applicable	
Describe **inclusion/exclusion criteria** if samples or animals were excluded from the analysis. Were the criteria pre-established?<br><br>If sample or data points were omitted from analysis, report if this was due to attrition or intentional exclusion and provide justification.	Not Applicable	
For every figure, are **statistical tests** justified as appropriate? Do the data meet the assumptions of the tests (e.g., normal distribution)? Describe any methods used to assess it. Is there an estimate of variation within each group of data? Is the variance similar between the groups that are being statistically compared?	Yes	Methods, Supplemental, Figure Legends

Sample definition and in-laboratory replication	Information included in the manuscript?	In which section is the information available? (Reagents and Tools Table, Materials and Methods, Figures, Data Availability Section)
In the figure legends: state number of times the experiment was **replicated** in laboratory.	Yes	Figure Legends
In the figure legends: define whether data describe **technical or biological replicates**.	Yes	Figure Legends

Ethics

Ethics	Information included in the manuscript?	In which section is the information available? (Reagents and Tools Table, Materials and Methods, Figures, Data Availability Section)
Studies involving **human participants**: State details of **authority granting ethics approval** (IRB or equivalent committee(s), provide reference number for approval.	Not Applicable	
Studies involving **human participants**: Include a statement confirming that **informed consent** was obtained from all subjects and that the experiments conformed to the principles set out in the WMA Declaration of Helsinki and the Department of Health and Human Services Belmont Report.	Not Applicable	
Studies involving **human participants:** For publication of **patient photos**, include a statement confirming that consent to publish was obtained.	Not Applicable	
Studies involving experimental **animals**: State details of **authority granting ethics approval** (IRB or equivalent committee(s), provide reference number for approval. Include a statement of compliance with ethical regulations.	Yes	Methods, Supplemental
Studies involving **specimen and field samples:** State if relevant **permits** obtained, provide details of authority approving study; if none were required, explain why.	Not Applicable	

Dual Use Research of Concern (DURC)	Information included in the manuscript?	In which section is the information available? (Reagents and Tools Table, Materials and Methods, Figures, Data Availability Section)
Could your study fall under dual use research restrictions? Please check biosecurity documents and list of **select agents and toxins** (CDC): https://www.selectagents.gov/sat/list.htm	Not Applicable	
If you used a select agent, is the security level of the lab appropriate and reported in the manuscript?	Not Applicable	
If a study is subject to dual use research of concern regulations, is the name of the **authority granting approval and reference number** for the regulatory approval provided in the manuscript?	Not Applicable	

Reporting

The MDAR framework recommends adoption of discipline-specific guidelines, established and endorsed through community initiatives. Journals have their own policy about requiring specific guidelines and recommendations to complement MDAR.

Adherence to community standards	Information included in the manuscript?	In which section is the information available? (Reagents and Tools Table, Materials and Methods, Figures, Data Availability Section)
State if relevant guidelines or checklists (e.g., **ICMJE, MIBBI, ARRIVE, PRISMA**) have been followed or provided.	Yes	Methods, Supplemental
For **tumor marker prognostic studies,** we recommend that you follow the **REMARK** reporting guidelines (see link list at top right). See author guidelines, under 'Reporting Guidelines'. Please confirm you have followed these guidelines.	Not Applicable	
For **phase II and III randomized controlled trials**, please refer to the **CONSORT** flow diagram (see link list at top right) and submit the CONSORT checklist (see link list at top right) with your submission. See author guidelines, under 'Reporting Guidelines'. Please confirm you have submitted this list.	Not Applicable	

Data Availability

Data availability	Information included in the manuscript?	In which section is the information available? (Reagents and Tools Table, Materials and Methods, Figures, Data Availability Section)
Have **primary datasets** been deposited according to the journal's guidelines (see 'Data Deposition' section) and the respective accession numbers provided in the Data Availability Section?	Yes	Methods, Supplemental
Were **human clinical and genomic datasets** deposited in a public access-controlled repository in accordance to ethical obligations to the patients and to the applicable consent agreement?	Yes	Methods, Supplemental
Are **computational models** that are central and integral to a study available without restrictions in a machine-readable form? Were the relevant accession numbers or links provided?	Not Applicable	
If publicly available data were reused, provide the respective **data citations in the reference list**.	Yes	Reference List
